# The speed of tubule formation of two fijiviruses corresponds with their dissemination efficiency in their insect vectors

**DOI:** 10.1186/s12985-016-0632-1

**Published:** 2016-10-19

**Authors:** Dongsheng Jia, Yu Han, Xiang Sun, Zhenzhen Wang, Zhenguo Du, Qian Chen, Taiyun Wei

**Affiliations:** Fujian Province Key Laboratory of Plant Virology, Institute of Plant Virology, Fujian Agriculture and Forestry University, Fuzhou, 350002 Fujian People’s Republic of China

**Keywords:** *Rice black-streaked dwarf virus*, Southern rice black-streaked dwarf virus, Tubule formation, Dissemination efficiency, Latent period

## Abstract

**Background:**

*Rice black-streaked dwarf virus* (RBSDV) and Southern rice black-streaked dwarf virus (SRBSDV) are two closely related fijiviruses transmitted by the small brown planthopper (SBPH) and white-backed planthopper (WBPH), respectively. SRBSDV has a latent period 4 days shorter than that of RBSDV, implying a more efficient spread in insect vector. Currently, the mechanisms underlying this higher efficiency are poorly understood. However, our recent studies have implicated a role of virus induced tubular structures in the dissemination of fijiiruses within their insect vectors.

**Methods:**

Immunofluorescence labeling was performed to visualize and compare the dynamics of P7-1 tubule formation of the RBSDV and SRBSDV in their own vector insects and nonhost *Spodoptera frugiperda* (Sf9) cells.

**Results:**

Tubule formation of SRBSDV P7-1 was faster than that of RBSDV P7-1. For RBSDV, P7-1 formed tubules were observed at 3-days post-first access to diseased plants (padp) in SBPH. For SRBSDV, these structures were detected as early as 1 day padp in WBPH. Importantly, similar phenomena were observed when P7-1 proteins from the two viruses were expressed alone in Sf9 cells.

**Conclusions:**

Our research revealed a relationship between the speed of P7-1 tubule formation and virus dissemination efficiency and also supports a role of such tubular structures in the spread of reoviruses within insect vectors.

## Background

Many plant viruses are transmitted by insect vectors in a circulative propagative manner [1]. Normally, these viruses enter the alimentary canal of an insect vector feeding on the phloem tissues of an infected plant. Then, the virus infects gut epithelial cells and, after multiplication, moves to other insect tissues or organs. After circumventing various physical or immunological transmission barriers, the virus reaches the salivary glands, from which it can be introduced into a naïve host plant [2, 3]. The latent period of these viruses in insect vectors, which refers to the time required for them to circulate within an individual insect before they can be successfully inoculated into a host plant, varies greatly from several days to several weeks [1, 2]. Although there are obvious epidemiological and agricultural implications, viral or insect vector factors responsible for the variation of latent period remain poorly understood.

*Reoviridae* is a family of viruses characterized by a non-enveloped icosahedral virion encapsulating a genome composed of 10–12 segments of double stranded RNA [4]. The family consists of 15 genera, 3 of which, *Phytorevirus*, *Oryzavirus* and *Fijivirus*, respectively, are plant-infecting viruses [5–7]. All the plant-infecting reoviruses are transmitted by planthoppers or leafhoppers in a circulative propagative manner, and several of them represent major viral pathogens of the Gramineae in East Asia [3]. In recent years, we have tracked the dissemination route of several plant-infecting reoviruses in their insect vectors [8–11]. In brief, we revealed a considerable diversity of strategies employed by these viruses to exploit their insect vectors [12]. However, a common finding is that all of them seem to use virus induced tubular structures to move within their insect vectors. In the gut, the tubules protrude from the surface of an infected cell and extend out to the gut lumen or to the muscle cells by crossing the basal membrane [9, 13]. In all cases examined, the major protein components of the tubular structure were encoded by the virus [13–16]. Disruption of the tubule by silencing the corresponding tubule-forming gene using RNA interference diminished the ability of the virus to spread both in the insect body and in a vector cell monolayer derived from the insect vector [9, 13, 15].

*Rice black-streaked dwarf virus* (RBSDV) and Southern rice black-streaked dwarf virus (SRBSDV) are two closely related fijiviruses [17, 18]. However, they are transmitted by different insect vectors, the small brown planthopper (SBPH) and white-backed planthopper (WBPH), respectively and they differ greatly with respect to their latent period in the insect vector, with that of the later being 4 days shorter than that of the former [18–21]. Our previous data showed that both viruses use P7-1 to form tubular structures within their insect vectors and that the P7-1 tubules played a role in viral dissemination in the insect body [13, 14]. In this study, we compared the dynamics of P7-1 tubule formation of the two viruses. If P7-1 is important for virus movement within the insect vector and the movement plays a role in determining the latent period length, we would observe faster tubule formation for SRBSDV, which exhibits a shorter latent period.

## Methods and results

**Fig. 1:**
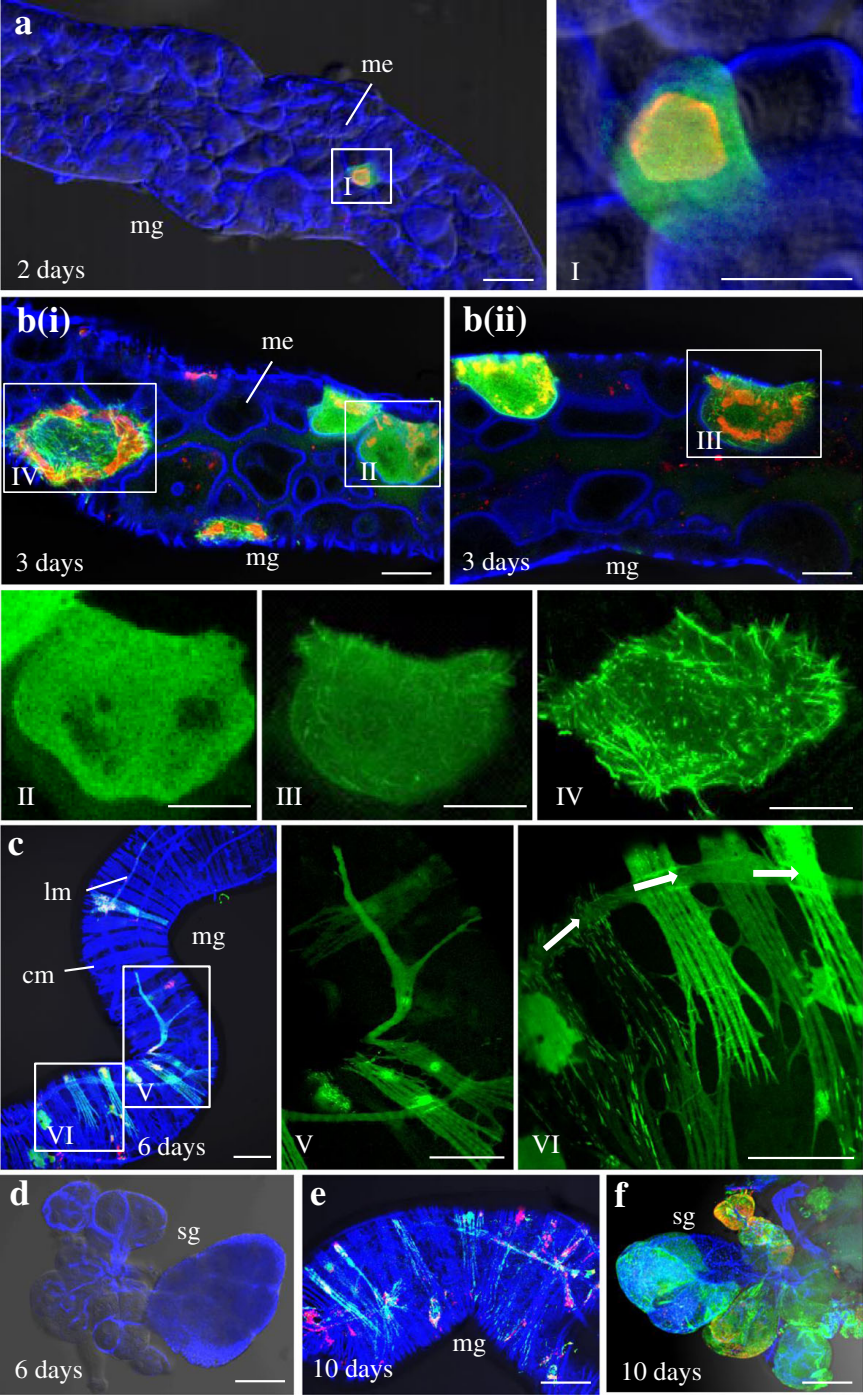
Immunofluorescence detection of *Rice black-streaked dwarf virus* (RBSDV) P7-1 in the alimentary canal of viruliferous small brown planthoppers (SBPHs). The internal organs of SBPHs were immunostained with P7-1-FITC (*green*), P9-1-rhodamine (*red*) and actin dye phalloidin-Alexa Fluor 647 carboxylic acid (*blue*) and then examined under confocal microscopy. **a** At 2 days post-first access to diseased plants (padp), P7-1 and P9-1 antigens had accumulated in midgut epithelial cells. Panel I is an enlarged image of the boxed area in panel **a**, showing P7-1 and P9-1 in epithelial cells. **b** By 3 days padp, P7-1 had formed into tubules in the midgut epithelial cells. Panels II, III and IV show enlarged images of the boxed areas II, III and IV in panels b(i) and b(ii), demonstrating the range of morphologies of P7-1 in the epithelial cells. **c** At 6 days padp, P7-1 and P9-1 antigens had accumulated in the circular muscle and longitudinal muscle of the midgut, but did not yet appear in (**d**) the salivary glands. Panels V and VI show enlarged images of the boxed areas V and VI in panel **c**, demonstrating the spread of P7-1 in the visceral muscle of the midgut. By 10 days padp, P7-1 and P9-1 antigens had accumulated in (**e**) the whole digestive system and (**f**) salivary glands. mg, midgut; hg, hindgut; sg, salivary gland; me, midgut epithelium; lm, longitudinal muscle, cm, circular muscle; scale bars, 30 μm

To investigate the dynamics of P7-1 tubule formation of RBSDV, 200 s-instar SBPHs were fed on RBSDV-infected rice plants (6.73 ± 1.64 × 10^11^ viral genome copies/μg rice RNA) for 2 days, and then placed on healthy rice seedlings grown at 26 °C, 16 h light/8 h dark and 70 % relative humidity. The internal organs of 30 insects were dissected at different time points to assess the accumulation and distribution of P7-1 by immunofluorescence microscopy as described previously [8]. At 2 days post-first access to diseased plants (padp), antigens of RBSDV P9-1, a major protein component of the reovirus replication factory called viroplasm [22], were immunolocalized in a small number of epithelial cells in the midgut of 32 % SBPHs (Table 1). At this time, P7-1 antigens were detected, revealing a diffuse distribution throughout the cytoplasm of the epithelial cells, suggesting that P7-1 protein expression began soon after RBSDV infection of the midgut cells (Fig. 1a). At 3 days padp, P9-1 and P7-1 antigens were still restricted to the epithelial cells of the midgut of 30 % SBPHs (Fig. 1b, Table 1). In some epithelial cells, tubular structures formed by P7-1 proteins were observed (Fig. 1b-III, b-IV). At 6 days padp, P7-1 proteins were detected in the external longitudinal and circular muscle fibers lining the midgut of 32 % SBPHs (Fig. 1c, Table 1). At this time, tubules were obviously detected (Fig. 1c-VI). However, a substantial proportion of the P7-1 proteins seemed to be diffusely distributed (Fig. 1c-V). P9-1 and P7-1 antigens were not found in the salivary glands (Fig. 1d, Table 1). These results suggested the time needed from tubule formation in epithelial to RBSDV movement to external tissues was about 3 days. At 10 days padp, P9-1 and P7-1 antigens were found to be predominately associated with the longitudinal and circular muscle fibers in the midgut. At this time, all P7-1 antigens were found to be associated with tubular structures (Fig. 1e). In addition, P9-1 and P7-1 antigens were also detected in salivary glands of 23 % SBPHs (Fig. 1f, Table 1). Interestingly, the dynamics of RBSDV P7-1 formed tubular structures corresponds with the latent period of this virus within its insect vector [20, 21].

**Table 1:** Occurrence of P7-1 in various tissues of vector at different days post-acquire virus as detected by immunofluorescence microscopy

Virus/vector	Days post-acquire virus	No. positive insects with P7-1 in different tissues (*n* = 30)								
		me			mm			sg		
		I	II	III	I	II	III	I	II	III
RBSDV/SBPH	2	10	8	11	0	0	0	0	0	0
	3	9	10	8	0	0	0	0	0	0
	6	8	7	8	10	8	11	0	0	0
	10	0	0	0	9	9	11	7	8	6
SRBSDV/WBPH	1	15	17	14	0	0	0	0	0	0
	2	22	23	20	0	0	0	0	0	0
	4	20	18	21	23	23	21	0	0	0
	6	0	0	0	22	20	23	15	13	16

*me* midgut epithelium, *mm* midgut muscle, *sg* salivary gland

**Fig. 2:**
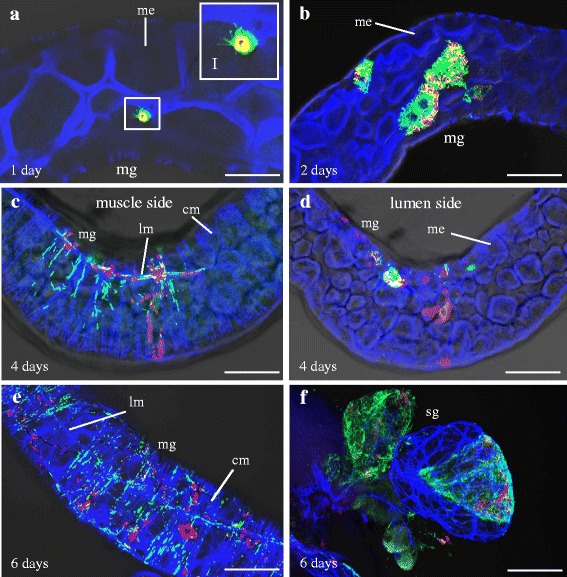
Immunofluorescent detection of Southern rice black-streaked dwarf virus (SRBSDV) P7-1 tubules in viruliferous white-backed planthoppers (WBPHs). The internal organs of viruliferous WBPHs at 1, 2, 4 and 6-day post-first access to diseased plants (padp) were immunolabeled with P7-1-FITC (*green*), P9-1-rhodamine (*red*) and the actin dye phalloidin-Alexa Fluor 633 carboxylic acid (*blue*) and then examined by confocal microscopy. **a** At 1 day padp, P7-1 and P9-1 antigens accumulated in midgut epithelial cells. Panel I is an enlarged image of the boxed area in panel A showing P7-1 and P9-1 in the epithelial cells. **b** By 2 days padp, more P7-1 and P9-1 antigens had accumulated in the midgut epithelial cells. **c** At 4 days padp, P7-1 and P9-1 antigens appeared in the circular muscle and longitudinal muscle of the midgut while (**d**) some P7-1 and P9-1 antigens were still accumulated in the epithelial cells of the midgut. By 6 days padp, P7-1 and P9-1 antigens had accumulated in (**e**) the whole digestive system and (**f**) salivary glands. mg, midgut; me, midgut epithelium; cm, circular muscle; lm, longitudinal muscle; sg, salivary gland; scale bars, 30 μm

To compare the spread of SRBSDV with that of RBSDV, 200 s-instar WBPHs were fed on SRBSDV-infected rice plants (8.69 ± 2.15 × 10^11^ viral genome copies/μg rice RNA) for 2 days and the dynamic localization of SRBSDV P9-1 and P7-1 was assessed in a similar manner. In contrast to the RBSDV observations, SRBSDV P9-1 and P7-1 antigens were detected as early as 1 day padp in epithelial cells of 51 % WBPH midgut, at which time P7-1 tubular structures were observed (Fig. 2a, Table 1). By 2 days padp, SRBSDV P9-1 and P7-1 had accumulated to higher levels, and P7-1 antigens were found to be associated with tubular structures in midgut of 72 % WBPHs (Fig. 2b, Table 1). At 4 days padp, as in the RBSDV experiment at 6 days padp, SRBSDV P9-1 and P7-1 antigens were associated with the longitudinal and circular muscle fibers in the midgut of 74 % WBPHs, and all P7-1 antigens were associated with tubular structures (Fig. 2c, Table 1), while some P9-1 and P7-1 antigens still appeared in epithelial cells (Fig. 2d). These results suggested SRBSDV needed about 2 days from tubule formation in epithelial cells to virus movement to external tissues. At 6 days padp, as in the RBSDV experiment at 10 days padp, all SRBSDV P9-1 and P7-1 antigens were associated with the longitudinal and circular muscle fibers in the midgut (Fig. 2e). Meanwhile, P9-1 and P7-1 antigens appeared in the salivary glands of 49 % WBPHs (Fig. 2f, Table 1). Again, the dynamics of SRBSDV P7-1 formed tubular structures corresponds with the latent period of this virus within its insect vector [18, 19].

**Fig. 3:**
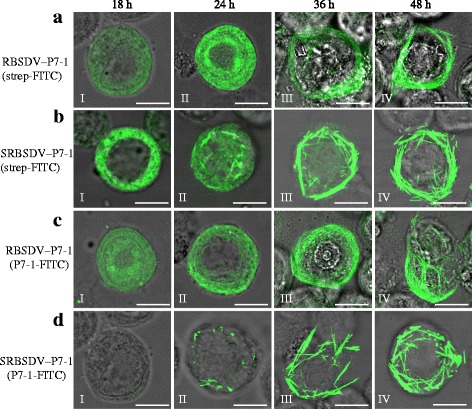
Nonstructural protein P7-1 fused with Strep-tag of *Rice black-streaked dwarf virus* (RBSDV) and Southern rice black-streaked dwarf virus (SRBSDV) expressed in Sf9 cells. At 18 (I), 24 (II), 36 (III) and 48 (IV) h post-inoculation, the recombinant (**a**) RBSDV and (**b**) SRBSDV P7-1 proteins fused with strep-tag were detected by immunofluorescence with a monoclonal anti-Strep-tag antibody and anti-mouse IgG conjugated with FITC. The recombinant (**c**) RBSDV and (**d**) SRBSDV P7-1 proteins fused with strep-tag were stained with RBSDV P7-1-FITC and SRBSDV P7-1-FITC, respectively. Scale bars, 5 μm

The above data suggested a faster tubule formation of SRBSDV than RBSDV. While for RBSDV, tubules were observed one day after the appearance of P7-1 antigens, for SRBSDV, there was no time lag detected between P7-1 accumulation and tubule formation. Interestingly, the faster tubule formation corresponded with the more rapid spread of SRBSDV from the gut to the salivary glands, which, likewise, corresponded with the shorter latent period of this virus in its insect vector. However, because the two viruses use two different insect vectors, there are multiple explanations for this observation. To distinguish among these possibilities and to further clarify the role of P7-1 in this process, we utilized the ability of P7-1 to form tubular structures when expressed alone in a heterologous system [23]. To accomplish this, RBSDV and SRBSDV P7-1 genes fused with Strep-tag were independently recombined into the Gateway baculovirus vector pDEST8, and the recombinant proteins were expressed in nonhost *Spodoptera frugiperda* (Sf9) cells in the presence of Cellfectin (Invitrogen) as described previously [23]. The cells were fixed and stained with the monoclonal anti-Strep-tag and fluorescein isothiocyanate anti-mouse immunoglobulin G, at various time points as described previously [23]. Interestingly, the faster P7-1 tubule formation of SRBSDV was recapitulated in this system. At 18 h post-inoculation (p.i.), the P7-1-strep of RBSDV diffusely distributed throughout the cytoplasm (Fig. 3a-I). The accumulation of P7-1-strep increased at 24 h p.i. At this time, it seemed that P7-1-strep appeared to have moved to the cell periphery (Fig. 3a-II). However, no tubular structure associated with P7-1-strep was observed until 36 h p.i. (Fig. 3a-III). At 48 h p.i., P7-1-strep was predominately associated with tubular structures (Fig. 3a-IV). Similar to that of RBSDV, SRBSDV P7-1-strep was abundantly expressed at 18 h p.i. However, unlike that of RBSDV, the fluorescence associated with SRBSDV P7-1 was more densely distributed (Fig. 3b-I). By 24 h p.i., some of the SRBSDV P7-1-strep proteins had formed observable tubular structures (Fig. 3b-II). Almost all of the SRBSDV P7-1-strep had assembled into tubular structures by 36 h p.i. (Fig. 3b-III). At 48 h p.i, all P7-1-strep signals of SRBSDV were associated with tubular structures (Fig. 3b-IV). Thus, P7-1 of SRBSDV has an intrinsic ability to form tubular structures faster than RBSDV. In addition, RBSDV P7-1 showed a diffuse distribution in the cytoplasm when it was first detected in SBPH epithelial cells. However, the diffuse distribution was not observed for SRBSDV P7-1 in WBPH epithelial cells, although it was observed when the SRBSDV P7-1 was expressed in sf9 cells (detected by monoclonal anti-strep-tag). To clarify this confusion, P7-1-FITC antibodies of RBSDV and SRBSDV were used to stain the corresponding P7-1-strep proteins expressed in Sf9 cells. Interestingly, diffuse distribution of RBSDV, but not SRBSDV P7-1, was observed at 18 and 24 h pi (Fig. 3c, d). This indicated that SRBSDV P7-1 antibodies may specifically recognize the tubular structures formed by this protein.

## Conclusion

Overall, our data established a relationship between the speed of P7-1 tubule formation and the spread efficiency of two fijiviruses within their insect vectors. This further confirmed a role of the tubular structures that we have observed across all studies of the viral dissemination of reoviruses within insect vectors [9, 13, 16]. The efficiency of viral dissemination from the gut to the salivary gland affects vector competence and plays a role in determining the latent period length of a virus within its insect vector (Figs. 1 and 2). As the latent period length has apparent epidemiological implications, it is a promising target for manipulating pathogens associated with vector-borne diseases. However, viral or vector factors that affect latent period length have thus far been elusive [24–26]. Accordingly, our study may represent one of the first steps towards understanding and exploring this biologically important property. The lack of an infectious clone system for inoculation of plant-infecting reoviruses prevented us from testing our hypothesis by swapping the P7-1 of the two fijiviruses. However, the P7-1 of the two fijiviruses share as much as 79 % of their amino acid sequence identity. Further studies should aim to identify amino acids or domains responsible for the biochemical or biophysical divergence of the P7-1 proteins of the two fijiviruses.

## Abbreviations

p.i.: Post-inoculation
padp: Post-first access to diseased plants
RBSDV: *Rice black-streaked dwarf virus*
SBPH: Small brown planthopper
Sf9: *Spodoptera frugiperda*
SRBSDV: Southern rice black-streaked dwarf virus
WBPH: White-backed planthopper
